# Lymphovascular Tumoral Emboli in Inflammatory Breast Cancer Result from Haptotaxis-Mediated Encircling Lymphangiogenesis

**DOI:** 10.3390/lymphatics2040016

**Published:** 2024-10-08

**Authors:** Justin Wang, Robert M. Hoffman, Yin Ye, Jordan Dillard, Sanford H. Barsky

**Affiliations:** 1 Scripps Mercy Hospital, MER 35, San Diego, CA 92103, USA; 2 AntiCancer, Inc., 7917 Ostow St., Suite B, San Diego, CA 92111, USA; 3 The Department of Surgery, University of California at San Diego, 9300 Campus Point Drive, #7220, San Diego, CA 92037, USA; 4 Department of Pathology, Anatomy and Cell Biology and the Clinical and Translational Research Center of Excellence, Meharry Medical College, 1005 Dr. D.B. Todd Jr. Boulevard, Nashville, TN 37208, USA

**Keywords:** lymphovascular tumor emboli, IBC, Mary-X, EMT, spheroidgenesis, encircling lymphangiogenesis, GFP-, RFP-, CFP-, and nestin-GFP transgenic reporter mice, haptotaxis, chemotaxis

## Abstract

Inflammatory breast cancer (IBC) is characterized by numerous tumor emboli within lymphatics. In a recent study, we observed tumor embolic budding both in vitro and in vivo within lymphovascular spaces and proposed this to account for the plethora of tumor emboli seen in IBC. These observations did not address, however, how lymphovascular invasion is initiated or the mechanisms involved. In the present study, using the well-characterized patient-derived xenograft (PDX), Mary-X, which exhibited florid lymphovascular invasion (LVI) in athymic mice (LVI) as defined by E-cadherin-positive tumor emboli within lymphatic channels distinguished by podoplanin and LYVE1 membrane and Prox1 nuclear immunoreactivities and spontaneous spheroidgenesis in vitro and human cases of IBC which showed similar LVI, we compared laser-captured microdissected emboli from Mary-X and from the cases of human IBC to non-embolic areas. Mary-X and IBC emboli expressed high levels of E-cadherin and no evidence of epithelial–mesenchymal transition (EMT). Mary-X spheroids expressed high levels of VEGF, especially VEGF-C, and stimulated both vascular and lymphatic endothelial haptotaxis. We then transplanted Mary-X serially into green, cyano, red, and nestin-green fluorescing protein (GFP-, CFP-, RFP-, and nestin-GFP) transgenic reporter mice in various combinations. Multicolor murine imaging studies indicated that reporter-labeled stroma initially encircled clumps of tumor cells and then served as a scaffold that supported nestin-GFP-labeled endothelial haptotaxis resulting in encircling lymphangiogenesis, confirmed by dual LYVE1 immunofluorescence. The present studies demonstrate a possible mechanism of a critical step of the tumor emboli formation of IBC.

## 1. Introduction

Lymphovascular invasion (LVI) or the formation of tumor emboli within lymphovascular spaces may be a critical step in the generation of micrometastasis and has been appropriately called “a metastasis caught in the act” [1–3]. Recent experimental evidence has suggested that these lymphovascular tumor emboli may give rise to circulating tumor cells (CTCs) which eventually result in metastases [4]. However, whether these experimental observations can be applied to only IBC or more widely to other types of breast cancer exhibiting LVI remains unclear in clinical studies [5].

Although most types of human cancer exhibit lymphovascular invasion at a given stage, the cancer that exhibits the most exaggerated degree of lymphovascular invasion is a type of breast cancer called inflammatory breast cancer (IBC). IBC specifically exhibits the florid lymphovascular invasion of adjacent dermal lymphatics whose obstruction contributes to overlying skin erythema, swelling, and tenderness [6,7].

Recently, our laboratory has exploited a patient-derived xenograft (PDX) model of IBC, termed Mary-X, which our laboratory established in previous studies [2–4,8,9] to show that the plethora of lymphovascular tumor emboli in IBC may be the result of geometric budding [4]. However, these observations do not explain how the first lymphovascular tumor embolus forms or how the tumor cells gain access to the lymphovascular network in the first place and the mechanisms involved.

The hypotheses which are dominant in the field are that invasion and metastasis including the formation of tumor emboli within lymphovascular spaces are manifestations of epithelial–mesenchymal transition (EMT) where tumor cells become motile, lose epithelial adhesion molecules, express matrix metalloproteinases, and invade adjacent stromal tissues with included lymphovascular spaces [10,11]. Synchronous with the process of EMT is the acquisition of a stem cell phenotype [12].

We used our Mary-X PDX model and microdissected lymphovascular tumor emboli from IBC cases to determine the mechanism of the initial steps of lymphovascular invasion and the formation of IBC emboli. We specifically investigated EMT, examined in vitro lymphovascular growth factor levels and effects on both vascular and lymphatic haptotaxis, and provided in vivo visual evidence through multicolor imaging studies in mice expressing spectrally distinct reporter genes.

## 2. Results

### 2.1. Formation and Characterization of IBC Emboli in Lymphatic Vessels

**Figure 1.**
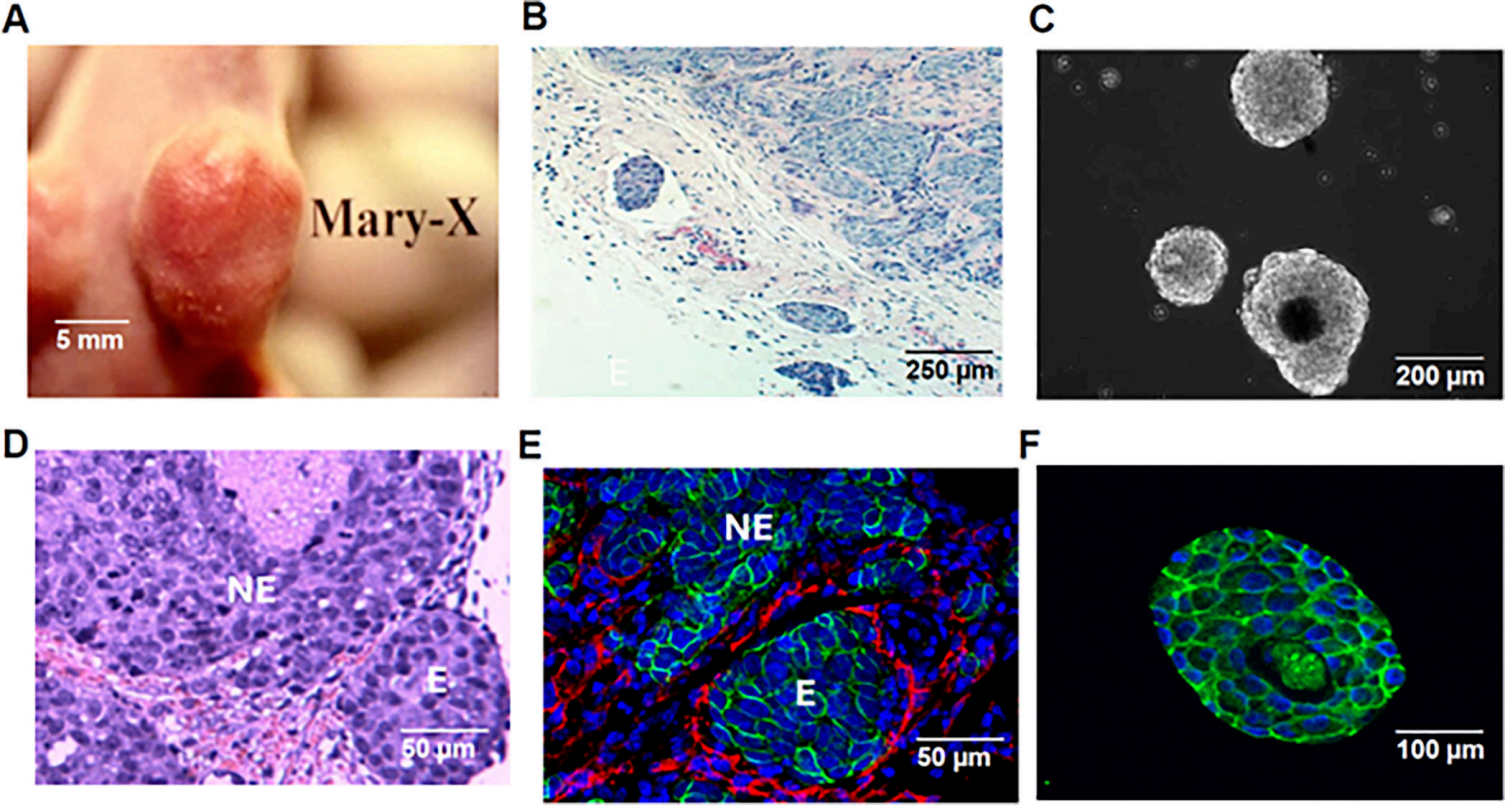
Mary-X in vitro and in vivo. The classic appearance of IBC is exhibited by Mary-X of overlying skin erythema (**A**) due to florid lymphovascular tumor emboli, especially in adjacent dermal lymphatics (**B**). An extirpated Mary-X PDX gave rise to spontaneously forming spheroids in suspension culture as observed by phase contrast microscopy (**C**). Mary-X contains non-embolic areas (NE) (upper center) juxtaposed to embolic areas (E) (right lower) (**D**) distinguished by absent and present circumferential podoplanin immunoreactivity (red immunofluorescence) in non-embolic (NE) vs. embolic (E) areas, respectively. Strong membrane E-cadherin immunoreactivity (green immunofluorescence) was present in both embolic (E) and non-embolic areas (NE) (**E**) and in the formed spheroids (**F**). Scale bars are provided.

Mary-X grew in athymic (nude) mice with its characteristic clinical signature of overlying skin erythema (Figure 1A) which is the result, as it is in humans, of florid lymphovascular tumor emboli (Figure 1B). When this PDX was extirpated and minced in culture, it spontaneously formed high-density spheroids (Figure 1C). Our previous studies demonstrated the transcriptome equivalence of these spheroids with the lymphovascular emboli of Mary-X [8]. Mary-X grew in a nodular fashion with islands of cancer cells embedded within a murine fibrovascular matrix (Figure 1D). In double-label immunocytochemical experiments with podoplanin (D2–40) which distinguished lymphatics from blood vessels (CD31), the vast majority (>90%) of vascular structures containing tumor emboli were lymphatics (Figure 1E). Both the embolic and non-embolic areas of Mary-X could, therefore, be clearly defined and both areas expressed strong E-cadherin tumor membrane immunoreactivity (Figure 1E). The Mary-X spheroids in vitro also exhibited strong E-cadherin immunoreactivity (Figure 1F).

**Figure 2.**
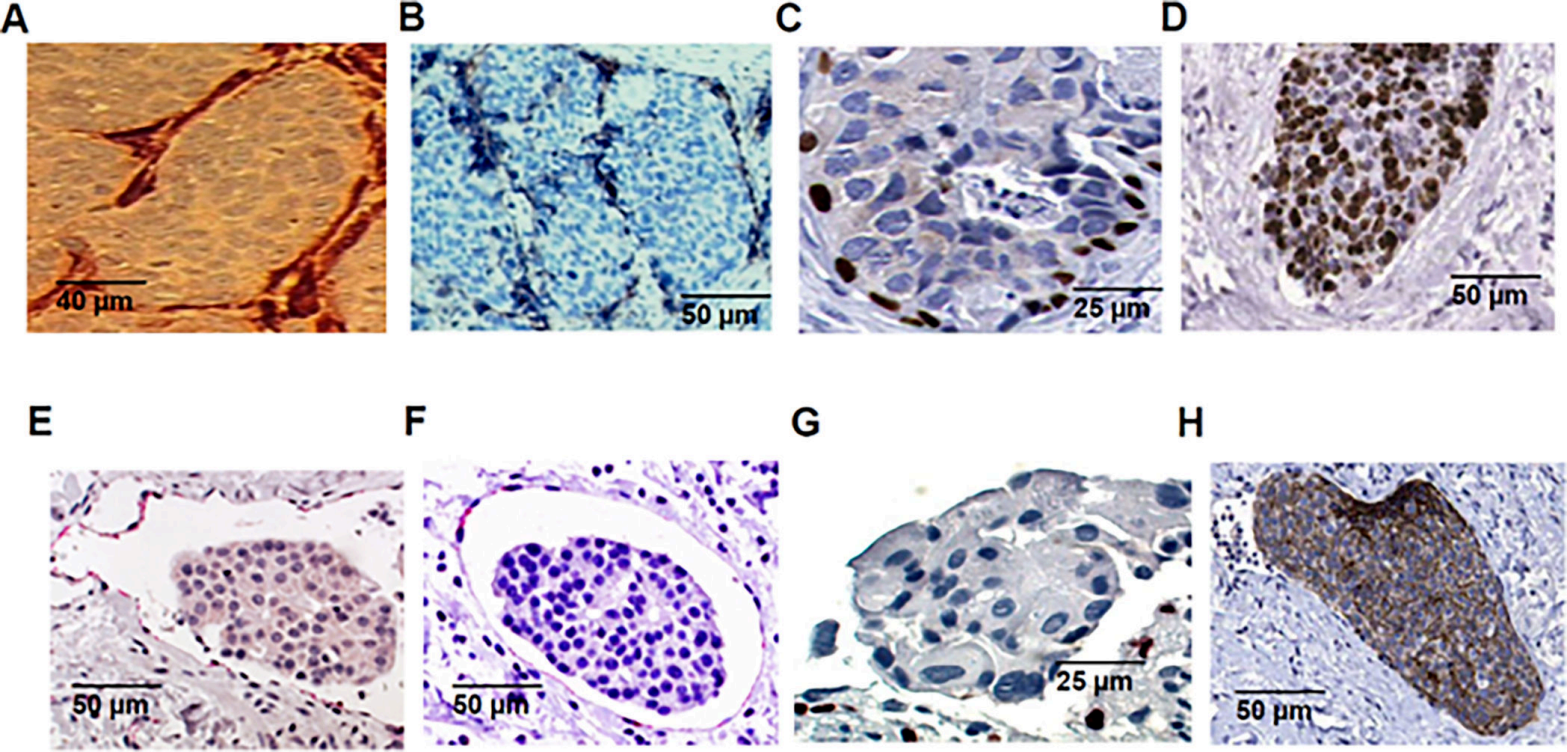
Mary-X and IBC in vivo. The florid tumor emboli in Mary-X are indeed within lymphatic channels confirmed by (**A**) membrane podoplanin, (**B**) membrane LYVE1, and (**C**) nuclear Prox1 immunoreactivity. The Mary-X tumor emboli show a higher level of Ki-67 proliferative immunoreactivity compared to the adjacent stroma (**D**). The cases of human IBC similarly show tumor emboli within lymphatic channels also confirmed by (**E**) membrane podoplanin, (**F**) membrane LYVE1, and (**G**) nuclear Prox1 immunoreactivity. The human IBC tumor emboli show a high level of membrane E-cadherin immunoreactivity (**H**). Scale bars are provided.

The observations that the vast majority of tumor emboli in Mary-X fall within lymphatic channels were confirmed by companion podoplanin, LYVE1, and Prox1 immunocytochemical studies (Figure 2A–C). In the cases of human IBC, similar membrane podoplanin, membrane LYVE1, and nuclear Prox1 immunoreactivities were observed surrounding the tumor emboli (Figure 2E–G). Similarly, in the cases of human IBC, strong tumor membrane E-cadherin immunoreactivity was observed both within the lymphovascular tumor emboli (Figure 2H) and within the non-embolic regions as demonstrated in previous studies [3]. In Mary-X, the tumoral nodules exhibited a high nuclear Ki-67 proliferative index which was not observed in the adjacent stroma (Figure 2D). The low proliferative stromal index reflected the fact that stromal cell migration rather than division mostly contributed to the stromal compartment.

### 2.2. In Vivo Embolic vs. Non-Embolic Comparisons of E-Cadherin Expression

**Figure 3.**
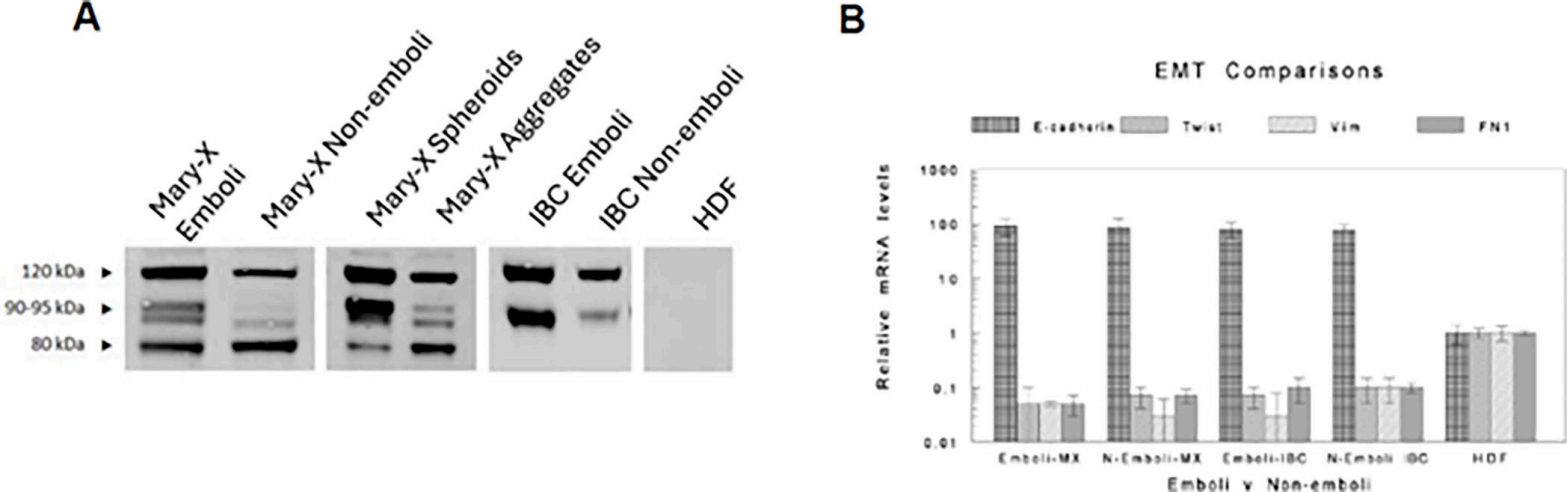
Western blot of E-cadherin and EMT comparisons. In the emboli vs.non-emboli of both Mary-X and the cases of IBC, strong E-cadherin expression was present (**A**). In addition, the proteolytic fragments of E-cadherin were more prominent in the emboli (**A**) but there was no overall decrease in E-cadherin expression. The relative mRNA levels compared to the reference levels in human dermal fibroblasts (HDFs) showed similarly significant increases in E-cadherin and similarly significant decreases in EMT-related genes (Twist, Vimentin, and FN1) equally in both emboli and non-emboli derived from Mary-X and the cases of IBC (**B**). The results depicted are the mean ± standard deviation of 5 separate experiments. Each experiment also had five separate replicates.

We compared the laser-captured microdissected emboli derived from Mary-X and cases of IBC to their respective non-embolic areas to investigate EMT with both Western blot and the real-time RT-PCR of the selected genes. The increases and decreases in the genes characterizing EMT are relative to a cell line (HDFs, human dermal fibroblasts) that expresses a non-epithelial fully mesenchymal phenotype, i.e., EMT fully transitioned to mesenchymal transcription. The genes assayed are the classic genes that are thought to initiate and characterize EMT. Both the embolic and non-embolic regions of both Mary-X and the cases of IBC showed strong expression of E-cadherin (Figure 3A). The embolic areas differed from the non-embolic areas in terms of the increased expression of a calpain-mediated proteolytic fragment of E-cadherin, E-cad/NTF1 (Figure 3A), which we had previously described [8]. There was no evidence of EMT, however, when the emboli were compared to non-embolic areas by real-time RT-PCR (Figure 3B).

### 2.3. In Vitro Lymphovasculogenesis Growth Factor Comparisons

**Figure 4.**
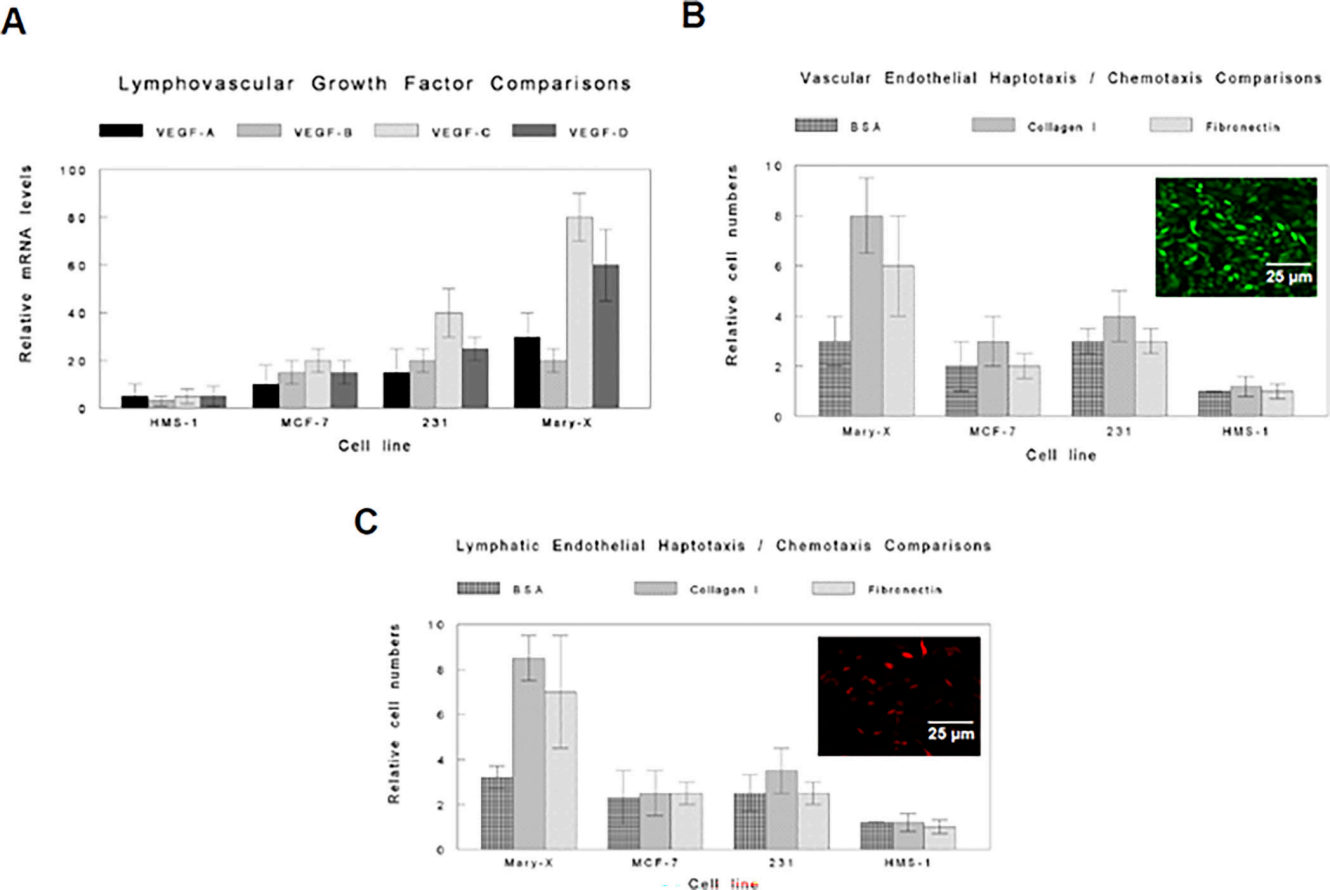
Lymphovascular growth factor and endothelial haptotaxis comparisons. The Mary-X spheroids compared to the induced spheroids of the other breast carcinoma lines showed significantly increased mRNA levels in VEGF-C (*p* < 0.01) and VEGF-D (*p* < 0.05) (**A**). The CM of the Mary-X spheroids compared to the CM of the induced spheroids of the other breast carcinoma lines showed significant stimulation of both vascular endothelial haptotaxis (**B**) and lymphatic endothelial haptotaxis (**C**) with either collagen I or fibronectin in both instances (*p* < 0.01; *p* < 0.05). No differential effects on chemotaxis (BSA control) were observed in either instance (*p* > 0.1). Vascular endothelial cells were confirmed as such by Alexa Fluor 488-conjugated goat anti-rabbit to rabbit anti-human CD31 [Inset] (**B**) and lymphatic endothelial cells were confirmed as such by Alexa Fluor 594-conjugated goat anti-rabbit to rabbit anti-human LYVE1 [Inset] (**B**). Scale bars are provided. For both the growth factor and endothelial haptotaxis comparisons, the levels were compared to a reference line, HMS-1, derived from HMS-X, a human myoepithelial xenograft (Table 1) [13,14].

The real-time RT-PCR of the relative mRNA levels of the VEGF family (VEGF-A, VEGF-B, VEGF-C, and VEGF-D) for the Mary-X spheroids vs. the induced spheroids of other breast carcinoma cell lines revealed that the Mary-X spheroids expressed higher levels of both VEGF-C (*p* < 0.01) and VEGF-D (*p* < 0.05) compared to the induced spheroids of the other breast carcinoma cell lines (Figure 4A). The other breast cancer cell lines used included the ER+, E-cadherin + MCF-7 line, and the ER−, E-cadherin−, MDA-MB-231 cell line. These lines were chosen for comparative studies with the ER−, E-cadherin + Mary-X because, when grown as xenografts, the MCF-7 and the MDA-MB-231 lines did not manifest lymphovascular invasion or tumor emboli formation within lymphovascular spaces. HMS-1 was used as a control cell line (Table 1) [13,14].

### 2.4. In Vitro Vascular and Lymphatic Endothelial Haptotaxis/Chemotaxis Comparisons of Mary-X Spheroid Conditioned Media (CM) vs. Other Breast Carcinoma Lines

The Mary-X spheroid CM stimulated increased vascular and lymphatic endothelial haptotaxis but not chemotaxis when compared with the CM of the other breast carcinoma lines (Figure 4B,C). The differential effect on endothelial haptotaxis was slightly greater when collagen I vs.fibronectin was used as the substrate (membrane coating) for both vascular and lymphatic haptotaxis (Figure 4B,C). There was no difference in the degrees of vascular vs.lymphatic haptotaxis stimulated by the Mary-X spheroid CM (*p* > 0.1). HMS-1 CM was used as a control (Table 1) [13,14].

### 2.5. In Vivo/In Vitro Spectrally Distinct Transgenic Reporter Gene Studies

**Figure 5.**
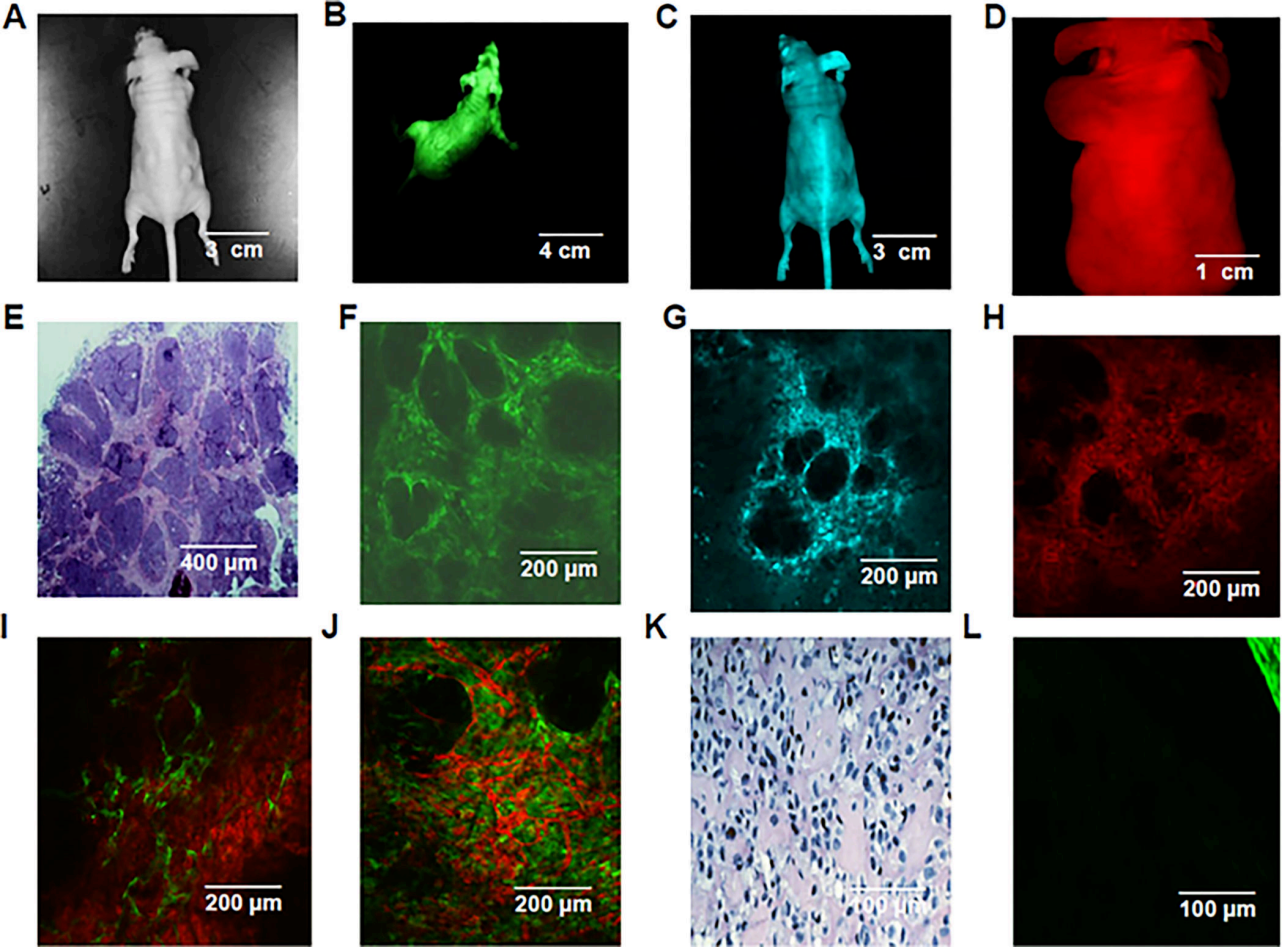
Multicolor murine imaging studies of Mary-X in transgenic reporter mice. Background athymic (**A**), GFP transgenic (**B**), CFP transgenic (**C**), and RFP transgenic (**D**) fluoresce appropriately. The extirpation of Mary-X and sectioning reveal the appearance of Mary-X in background mice (hematoxylin and eosin) (**E**) and the respective transgenic mice (**F**–**H**). The Mary-X tumor nodules do not fluoresce but are surrounded by their respective transgenic stromal fluorescence (**F**–**H**). Mary-X transplanted to RFP transgenic mice followed by transplantation to GFP transgenic mice (**I**) and vice versa (**J**) showed an admixture of fluorescent signals reflecting both GFP and RFP mesenchymal transgenic contributions. HMS-X [13,14], which secretes a human extracellular matrix devoid of murine stroma, lymphatics, and blood vessels (**K**) predictably contains no transgenic reporter fluorescence except around its periphery which represents murine stroma and vessels (right upper) (**L**). Scale bars are provided.

Athymic mice with spectrally distinct reporter transgenes, GFP-, RFP-, and CFP- [15–19], constitutively fluoresced their respective colors (Figure 5A–D). The serial transplantations of unlabelled Mary-X in groups of 10 mice revealed that the murine stroma around the Mary-X nodules fluoresces their respective transgene (Figure 5E–H) and that their mesenchymal stroma carries over with serial passage from mouse to mouse (Figure 5I,J). These observations were observed irrespective of the order of transplant. In contrast, a control PDX derived from a matrix-secreting human benign myoepithelial neoplasm, HMS-X exhibited no juxtaposed fluorescing murine stroma (Figure 5K,L) since it had been previously shown that its extracellular matrix was largely human in origin [13,14].

**Figure 6.**
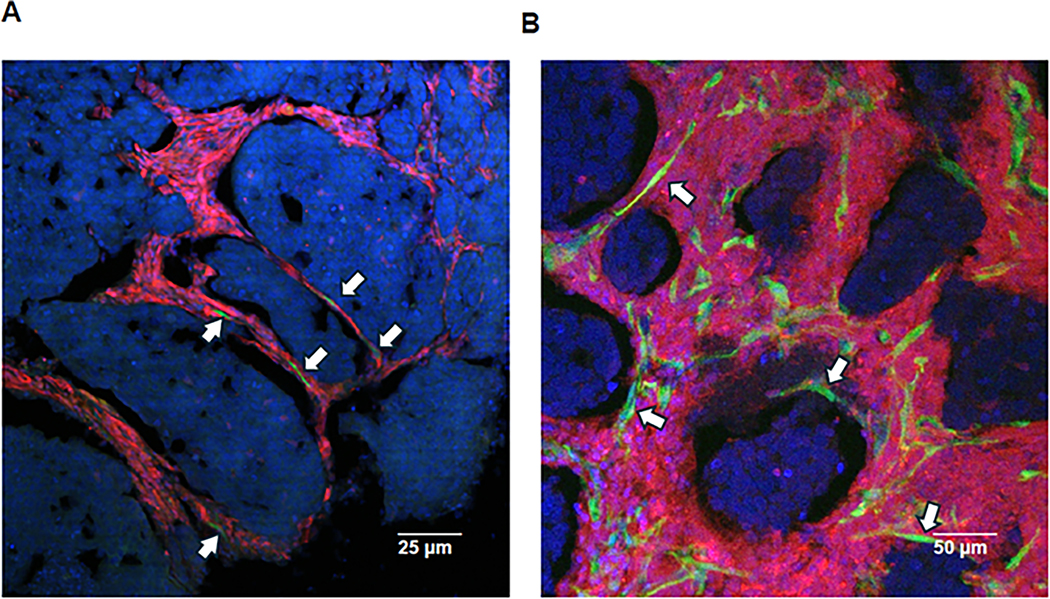
Multicolor murine imaging studies of Mary-X with serial transplantation into nestin-GFP transgenic mice. The extirpation of Mary-X sectioned and counterstained with DAPI reveals positive tumor clumps surrounded by RFP reporter mesenchyme and rare GFP-fluorescing endothelial cells (arrows) migrating along the RFP-labeled mesenchymal scaffold (1 month) (**A**) and then to encircle the tumor cell clumps by continued migration (arrows) along this scaffold (2 months) (**B**). Scale bars are provided.

**Figure 7.**
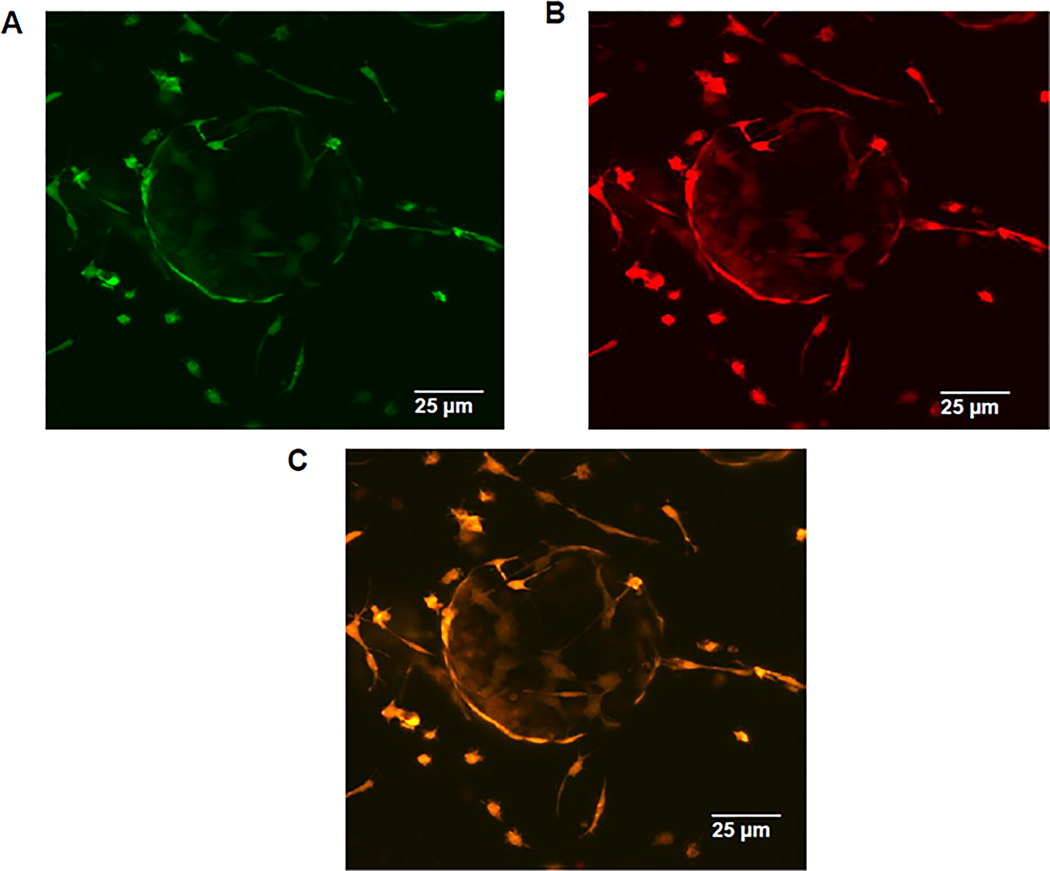
Multicolor imaging studies of Mary-X into nestin-GFP transgenic mice co-localized with lymphatic-specific markers. The extirpation of Mary-X (3–4 months) and sectioned reveals tumor clumps surrounded by fully encircling nestin-GFP green fluorescing cells migrating along the mesenchymal scaffold (**A**). Alexa Fluor 594-conjugated goat anti-rabbit to rabbit anti-mouse LYVE reveals colocalized red immunofluorescence (**B**) resulting in hybrid yellow fluorescence confirming that the encircling nestin-GFP fluorescing cells are indeed lymphatic in nature (**C**). Scale bars are provided.

In the serial passages of Mary-X first into RFP reporter transgenic mice and subsequently passed into nestin-GFP transgenic mice which express GFP in nascent vessels [20], rare GFP-fluorescing endothelial cells were observed after one month to be migrating along an RFP-labeled mesenchymal scaffold and then after two months to nearly surround the DAPI fluorescing emboli of Mary-X (Figure 6A,B). The order of serial transplant was important because this distinct pattern of fluorescence was not observed if the order of serial transplantation was reversed, indicating that in order to visualize the endothelial haptotaxis and the encircling lymphangiogenesis, the endothelial fluorescence had to be seen against a pre-existing color-contrasting mesenchymal background. To confirm that the nestin promoter gene was being expressed in lymphatic endothelial cells as opposed to either vascular endothelial cells, myoepithelial cells, or fibroblasts, we carried out co-labeled immunofluorescence studies using a red fluorescing Alexa Fluor 594-conjugated anti-LYVE1 on the Mary-X sections containing the nestin-GFP green fluorescing cells and demonstrated dual hybrid fluorescence (Figure 7A–C). In these studies, we used Mary-X xenografts that had been growing for 3–4 months in nestin-GFP transgenics to allow for the encircling lymphangiogenesis to complete. On the basis of these latter experiments, we believe that the fluorescent GFP signals observed in the nestin-GFP mice reflect lymphatic endothelial haptotaxis and encircling lymphangiogenesis.

## 3. Discussion

PDX mouse models have emerged as more reliable models of individualized cancer therapy, drug development, and coclinical trials than cell line-derived xenograft and genetically engineered mouse models [6,21] and yet many PDX models do not adequately replicate the totality of biology that their tumors of origin exhibit in patients. In IBC, for example, although a number of PDXs have been established, most do not exhibit the exaggerated phenotype of lymphovascular invasion that patients with the disease exhibit [15,16].

The present study is the first to demonstrate the establishment of lymphatic emboli in real time by visualizing the fate of labeled cancer cells in lymphovascular spaces in vivo using transgenic mice with spectrally distinct reporter genes. The lymphatic nature of the spaces was confirmed by dual fluorescence studies with lymphatic-specific biomarkers.

Since the conventional thinking of lymphovascular invasion has regarded this step as an extension of tumor invasion that involves EMT [10–12,17–19], we first decided to address this possible mechanism by comparing tumor emboli from Mary-X and the cases of IBC with their respective non-embolic areas. Both emboli and non-emboli expressed high levels of E-cadherin with the increased generation of E-cad/NTF1 within the emboli. We have reported calpain 2-mediated E-cadherin proteolysis generating E-cad/NTF1 in previous studies [4,8]. In the present study, we extended these observations to investigate the presence of EMT per se and found no evidence for it. One could argue that EMT is still occurring within minor subpopulations of the non-embolic areas and is immediately followed by mesenchymal–epithelial transition (MET) within the emboli. The same reasoning could be applied to the budding emboli where E-cadherin overexpression was also observed [4].

Interestingly, EMT in cancer progression has also been linked to the acquisition of a stem cell phenotype [12]. Yet, our previous studies have shown that Mary-X already expresses a stem cell phenotype in both its proliferating population and its aldehyde dehydrogenase-positive stem cell subpopulation [22]. Hence, Mary-X is already stem cell in nature without ever having to undergo an EMT.

Since our initial observations for the genesis of the lymphovascular embolus in IBC did not support EMT and the direct invasion of the pre-existing lymphovascular spaces, we investigated in the present study whether Mary-X might be stimulating new lymphangiogenesis. We, therefore, searched for evidence that Mary-X was producing lymphovascular growth factors and found, in fact, that Mary-X expressed high levels of VEGF-C and VEGF-D, two factors that have been implicated in the growth and proliferation of lymphatics [23].

Because the growth and proliferation of lymphatics per se, while necessary, was not sufficient to explain the genesis of the lymphovascular tumor embolus within lymphatic spaces, we also searched for evidence that Mary-X might be selectively stimulating endothelial haptotaxis [24,25] as well and found that it was. In fact, endothelial haptotaxis seemed to be a much more important phenomenon than endothelial cell division as the Ki-67 proliferative index of the stroma was low.

Since the formation of tumor emboli within lymphovascular spaces is an in vivo phenomenon, we decided to use transgenic reporter mice which allowed us to investigate the interactions between the human tumor cells and the murine extracellular matrix. A number of previous studies have used these reporter gene transgenic mice both singularly and in combination to visualize important PDX paracrine interactions [26–30]. These previous studies with mice with reporter genes had clearly demonstrated that the explanted xenograft not only acquires the stromal cells of the host but that these stromal cells carry over in the serial transplantation. We observed this same phenomenon in the Mary-X serial transplants. To our knowledge, however, our in vivo and ex vivo studies of Mary-X explants in transgenic reporter mice are the first to specifically examine the dynamic changes occurring within the surrounding extracellular matrix, specifically with respect to lymphatic endothelial haptotaxis.

Because we were interested in lymphovasculogenesis, the phenomenon raised by our in vitro studies, we decided to specifically utilize the nestin promoter-driven GFP mouse (nestin-GFP). This transgenic reporter mouse model has been used in a number of previous studies to investigate angiogenesis [31–33]. Some of these previous studies demonstrated that endothelial stem cells located within hair follicles express the reporter construct linked to the nestin promoter [32]. Other studies have demonstrated that nestin promoter-driven genes were also expressed within lymphatic endothelium [34]. We reasoned that since Mary-X contains numerous tumor emboli nearly all within lymphatic spaces, that using the nestin reporter mouse could shed light on the nature of this process. We observed that lymphatic endothelial cells expressing nestin-GFP could be visualized migrating along an RFP-expressing mesenchymal scaffold to encircle the tumor clumps, thereby supporting our hypothesis of endothelial haptotaxis and encircling lymphangiogenesis.

**Figure 8.**
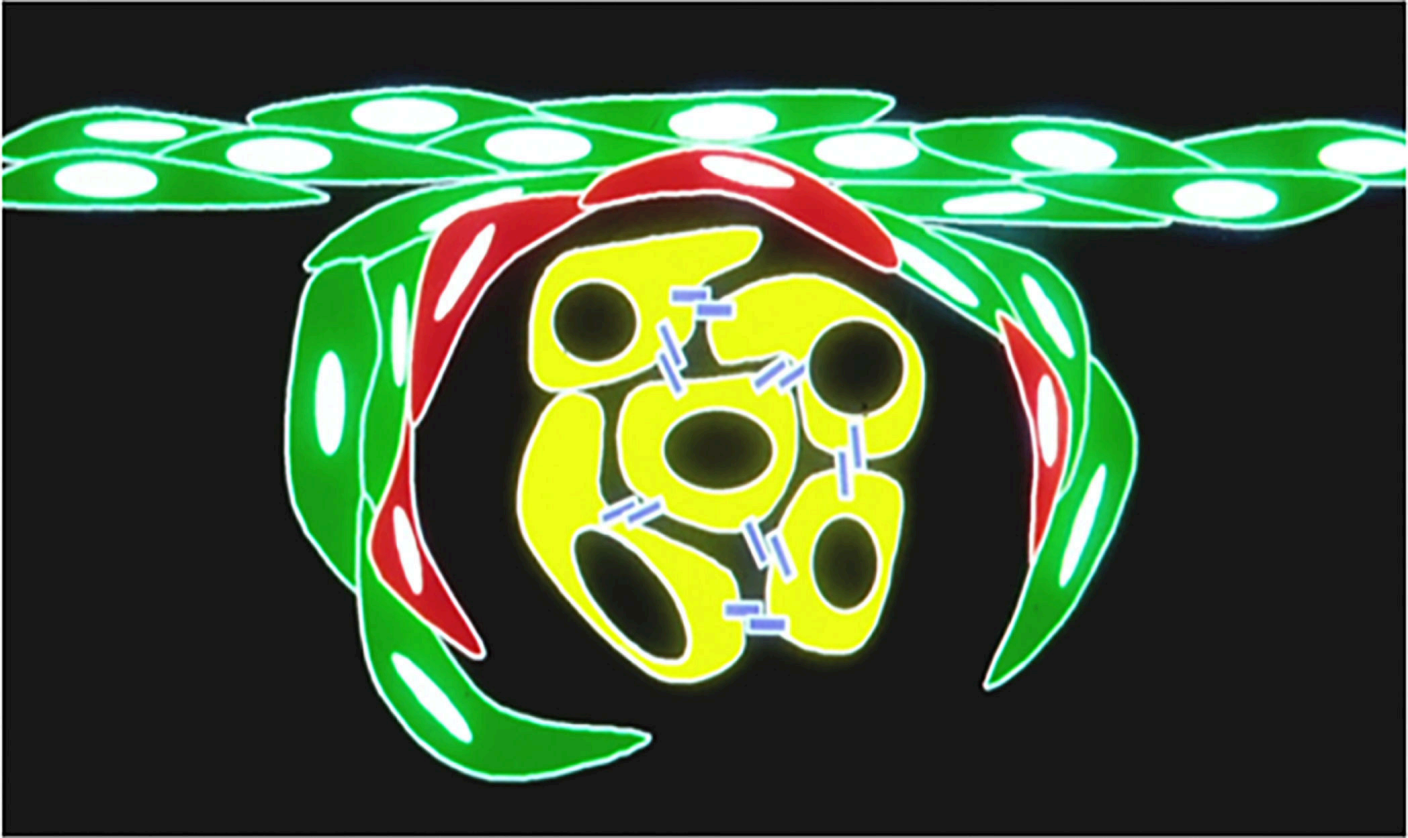
Schematic of endothelial haptotaxis resulting in encircling lymphangiogenesis. Migrating lymphatic endothelial cells (red) migrate along a mesenchymal scaffold (green) and encircle the tumor cell clumps (yellow) created by E-cadherin homodimers (blue). The clumps find themselves within a lymphatic space.

We, in an earlier study, had raised this hypothesis when we mixed Mary-X spheroids with murine embryonal fibroblasts and observed that the fibroblasts encircled the spheroids both in vitro and in vivo [35]. In the present study, we present more direct and compelling evidence that strengthens this hypothesis that IBC emboli are established in the process of encircling lymphovasculogenesis. This evidence includes the following: (1) in the case of Mary-X and IBC, our cumulative findings of the lack of EMT in the emboli compared to the non-emboli both in Mary-X and the human cases of IBC; (2) the strong VEGF growth factor, especially VEGF-C and VEGF-D compared to the other breast carcinoma cell lines; (3) the strong differential Mary-X spheroid CM effect specifically on both vascular and lymphatic endothelial haptotaxis; and (4) the transgenic reporter murine imaging studies of nestin-GFP-labeled endothelial haptotaxis along a pre-existing mesenchymal scaffold with the confirmatory dual fluorescence labeling of lymphatic specific markers. These four results suggest that lymphovascular tumor emboli are the result of encircling lymphangiogenesis (Figure 8).

It is important to mention the possibility that although the florid lymphovascular emboli in our PDX model may generate high levels of CTCs and pulmonary metastases, the relationship of lymphovascular tumor emboli to overall metastasis in general has not been proven and is somewhat controversial. Although there is some evidence suggesting that LVI contributes to local (or locoregional) recurrence in animal models [36,37], perhaps by creating a reservoir or a lymphatic niche for long-term maintenance of tumor cells in proximity to the original tumor bed, there are very few data, either from pre-clinical or clinical research, consistent with a causative relationship between lymphatic and distant metastasis. For example, breast cancer patients with sentinel LN metastasis experienced no benefit, neither in terms of regional recurrence-free survival nor overall survival, from complete axillary LN dissection as compared to sentinel LN dissection only [38].

## 4. Materials and Methods

### 4.1. Institutional Approvals

The initial xenograft studies were conducted under UCLA’s Human Subject Protection Committee and the Chancellor’s Animal Research Committee (Certification 95–127-11). Continuing animal studies were approved by The Ohio State University’s Animal Care and Use Committee (IACUC), protocol 2007A0218 and by The Ohio State University’s Institutional Biosafety Committee, protocol 2007R0057. Additional animal studies were approved by the University of Nevada’s School of Medicine and the Nevada Cancer Institute’s IACUC, protocols 00439 and 00440 when the corresponding author of this study was affiliated with these previous institutions. The subsequent animal studies were conducted under an Interinstitutional Agreement between the California University of Science and Medicine and AntiCancer, Inc. using AntiCancer’s IACUC approval under NIH Assurance number, A3873. The final animal studies were carried out at Meharry Medical College, NIH Assurance number, A3420.

In total, 100 cases of IBC had been randomly selected from a database and The Ohio State University’s Information Warehouse and anonymized. A total of 25 cases of non-IBC showing prominent lymphovascular invasion were obtained from the Meharry Medical College and its Translational Pathology Shared Resource Core. We had previously created tissue microarrays (TMAs) from these cases embedded in paraffin and collected a limited number of banked fresh frozen material, de-identified with appropriate IRB approvals in place.

### 4.2. Emboli vs. Non-Emboli Comparisons

Tumor from each paraffin-embedded donor block was stained and scanned. Our specific imaging algorithms based on the Gaussian kernel and specific circumferential lymphovascular immunoreactivities demonstrated previously [39] were used.

#### Immunohistochemistry

The immunohistochemistry (IHC) studies utilized tumor proliferation (Ki-67) and adhesion (E-cadherin) markers and lymphovascular (podoplanin (D2–40), CD31, LYVE1, and Prox1 markers. The biotinylated primary antibodies used were as follows: Ki-67 (clone MIB-1, Dako catalog number M7240), E-cadherin (clone NCH-38, Dako catalog number M3612), D2–40 (clone D2–40, Dako, catalog number M3619), CD31(rabbit polyclonal, Spring Bioscience, catalog number E11114, Pleasanton, CA, USA), LYVE1 (Invitrogen, Waltham, MA, USA), and Prox1 (Molecular Depot, San Diego, CA, USA).

For the immunostaining methods (Ki-67, E-cadherin, D2–40, and CD31), the primary antibody was diluted at 1:100 and incubated for 30 min at room temperature. For Ki-67, E-cadherin, D2–40, LYVE1, and Prox1, the detection system used was a labeled Streptavidin–Biotin Complex. DAB chromogen was applied to develop the color. Alternatively, the detection system MACH 4 (Biocare Medical catalog M4U536L) was used with Vulcan Fast Red to develop the color. A total of 100 emboli from each IBC and 10 emboli from each non-IBC case were analyzed.

### 4.3. ATCC Patent Deposits and Cell Identification

Mary-X and its in vitro spheroids were deposited in the ATCC cell repository (Manassas, VA, USA) as PTA-2737 and PTA-27376, respectively, and recently verified and re-verified to be both novel and human in origin (STRA4993). Mary-X is a well-known PDX of inflammatory breast cancer and has been used in numerous previous studies [2–4,8,9]. Mary-X has been grown in either the mammary fat pad or the flank. The tumor’s histology, biomarker profile, and biology are independent of the orthotopic or non-orthotopic transplantation site.

### 4.4. Confocal Single and Double Label Immunofluorescence Experiments on Mary-X and Mary-X Spheroids

Mary-X spheroids and loose aggregates were subjected to single-label immunofluorescence studies using Alexa Fluor 488-conjugated 24E10 (#3199) (Cell Signaling Technology, Inc., Danvers, MA, USA) which recognized both E-cad/FL and E-cad/NTF1. In order to immobilize the spheroids and loose aggregates, glass-bottom dishes were coated with Cell-TEK adhesive. After washing with PBS 4–5 times, each for 10 min, the spheroids and aggregates were incubated with 24E10.

Mary-X was also subjected to double-label immunofluorescence studies. The double-label immunofluorescence experiments were carried out using the following combinations of antibodies: Alexa Fluor 488-conjugated 24E10 which recognized both E-cad/NTF1 and E-cad/FL and goat polyclonal antibody to mouse podoplanin or CD31 (R&D Systems, Inc., Minneapolis, MN, USA) which recognized murine lymphatics or blood vessels followed by Alexa Fluor 594-conjugated donkey anti-goat (#A11058) (Invitrogen, Inc.). Tumor emboli were recognized by E-cadherin positivity surrounded by circumferential podoplanin (lymphatics) or CD31 (vessels) positivity.

Both the dishes and the sections were finally mounted with a Vectorshield mounting medium with DAPI (#H-1200) (Vector Laboratories) and viewed with an Olympus Fluoview-1000 confocal scanning system.

### 4.5. Laser Capture Microdissection

Both paraffin-embedded and frozen sections (8 μm) of Mary-X and human IBC cases were obtained, fixed in 70% ethanol, stained with hematoxylin, and progressively dehydrated. Tumor emboli and non-embolic solid areas of Mary-X and human IBC cases were microdissected using a Pixcell II Laser Capture Microdissection 788 Laboratory System (Arcturus, Inc., Mountain View, CA, USA) and stored at −80 °C. At least 100 emboli were obtained from each case and processed.

### 4.6. Preparation of Protein Lysates and Western Blot Analysis

The xenografts and laser-captured microdissected and pooled frozen IBC emboli and non-emboli were lysed using ice-cold RIPA lysis buffer (Pierce Biotechnology, Inc., Rockford, IL, USA). For Western blot analysis, boiled protein was loaded onto a 4–12% precast gradient gel (Invitrogen, Inc.), transferred to nitrocellulose membranes (Bio-rad, Hercules, CA, USA), and incubated with either E-cadherin rabbit mAb (24E10) or rabbit anti-human ectodomain E-cadherin (H108) (Santa Cruz Biotechnology, Santa Cruz, CA, USA) followed by anti-rabbit IgG, HRP-linked antibody (Cell Signaling Technology Inc., Danvers, MA, USA). Rabbit mAb (13E5) was used as a housekeeping protein, ACTB. Bound antibodies were detected by a chemiluminescent detection system (West Femto) (Pierce Biotechnology, Inc.) according to the manufacturer’s instructions.

### 4.7. In Vitro Studies with Mary-X Spheroids and Other Breast Cancer Cell Lines

Mary-X was placed in culture and gave rise to spontaneous spheroids in suspension culture [2–4]. The spheroids were seeded on 24-well plates in DMEM supplemented with 10% FBS.

Other breast cancer cell lines which included the E-cadherin positive (MCF7) and negative (MDA-MB-231) cell lines (ATCC, Manassas, VA, USA) were cultured in Dulbecco’s Modified Eagle’s Medium (DMEM) supplemented with 10% (vol/vol) FBS and 100 U/mL penicillin/streptomycin. These lines grew as monolayers but could be induced to grow as spheroids. A total of 5 × 10^4^ cells were seeded on 24-well Ultra-low Attachment (ULA) plates (Thermo Fisher Scientific, Waltham, MA, USA). Human dermal fibroblasts (HDFs), derived from human adult skin, were gifted by Andrew C. Issekutz, Dalhousie University, Halifax, Canada, and HMS-1 and HMS-X, a cell line and transplantable PDX derived from a human benign salivary gland myoepithelial tumor [13,14] were used as reference controls in their respective assays. Serum-free conditioned media (CM) from each of these samples was collected for 24–48 h and concentrated 10-fold with Amicon filtration (Millipore Sigma, Burlington, MA, USA)

### 4.8. EMT and Growth Factor Comparisons

Real-time RT-PCR was performed on both microdissected emboli vs. non-emboli from both Mary-X and the cases of IBC. The real-time RT-PCR was performed on an ABI 7500 Real-Time PCR System (Applied Biosystems, Inc., Foster City, CA, USA). cDNA was combined with primer sets and Power SYBR® Green PCR Master Mix (Applied Biosystems, Inc.) was used. The gene expression levels were calculated by using the 7500 System SDS software v1.4 (Applied Biosystems, Inc.).

The primer sets (forward and reverse) used for the EMT comparisons included the following: hEcad-5 TGCCCAGAAAATGAAAAAGG hEcad-3 GTGTATGTGGCAATGCGTTC; hTwist-5 GGAGTCCGCAGTCTTACGAG hTwist-3 TCTGGAGGACCTGGTAGAGG; hVim-5 GAGAACTTTGCCGTTGAAGC hVim-3 GCTTCCTGTAGGTGGCAATC; hFN1–5 CAGTGGGAGACCTCGAGAAG hFN1–3 TCCCTCGGAACATCAGAAAC. As a comparative control, HDF was used as a reference cell line.

Real-time RT-PCR was also performed on the spontaneous Mary-X spheroids and the induced spheroids of the other breast cancer cell lines used in the study. The primer sets (forward and reverse) used for the lymphovascular growth factor comparisons included the following: hVEGF-A, 5 CTTGCCTTGCTGCTCTACC-3′, 5′-CACACAGGATGGCTTGAAG-3′ hVEGF-B, 5′-AGCACCAAGTCCGGATG-3′, 5′-GTCTGGCTTCACAGCACTG-3′ hVEGF-C, 5′-TGCCGATGCATGTCTAAACT-3′, 5′-TGAACAGGTCTCTTCATCCAGC-3′ hVEGF-D, 5′-GTATGGACTCTCGCTCAGCAT-3′, 5′-AGGCTCTCTTCATTGCAACAG-3′. The housekeeping gene, hGAPDH-5 and primers: ACCCAGAAGACTGTGGATGG hGAPDH-3 TCTAGACGGCAGGTCAGGTC was used as RNA control. As a comparative cellular control, HMS-1 was used as a reference cell line.

The real-time RT-PCR experiments were repeated 5 times and the results of relative mRNA levels were depicted as mean ± standard deviation.

### 4.9. Vascular and Lymphatic Endothelial Haptotaxis/Chemotaxis Comparisons

Experiments were conducted to evaluate the effects of the concentrated CM of the Mary-X spheroids on endothelial haptotaxis and chemotaxis and compare this to the effects of CM of the other breast carcinoma cell lines. Both human umbilical vein endothelial cells (HUVECs) (ATCC, Manassas, VA, USA) and human lymphatic endothelial cells derived from juvenile foreskin (PromoCell, VWR, Lutterworth, Leicestershire, UK) were grown in media containing Endothelial Cell Growth Supplement (Fisher Scientific, Pittsburgh, PA, USA, cat# CB-40006). The identity and purity of each cell type were determined by rabbit anti-CD31 and rabbit anti-LYVE1 (Invitrogen, Waltham, MA, USA) immunofluorescence studies using Alexa FluorTM 488 and 594 goat anti-rabbit secondary antibodies, respectively (Invitrogen, Waltham, MA, USA). As before, the dishes were viewed with an Olympus Fluoview-1000 confocal scanning system.

The CytoSelect™ (Cell Biolabs, Inc., San Diego, CA, USA) 24-well cell haptotaxis/chemotaxis assay (8 μm, collagen I or fibronectin-coated or uncoated, both colorimetric and fluorometric formats) were used [24]. The assay uses polycarbonate membrane inserts (8 μm pore size), the bottom side of the insert coated with either collagen I or fibronectin (for haptotaxis) or uncoated (for chemotaxis). Briefly, 10^6^ endothelial cells were added to the upper well and CM to the lower well and incubated for 24 h After washing and swabbing the upper chamber, the cells which had migrated through the membrane were counted by staining or fluorescence.

### 4.10. In Vivo/In Vitro Transgenic Reporter Imaging Comparisons

To gain visual insight into the interactions between the cancer cells and the surrounding stroma occurring within the growing xenograft, Mary-X explants were serially transplanted into different reporter transgenic mice which included GFP-, CFP-, RFP-, and nestin-GFP transgenics [27–33]. Groups of 10 mice for each transgenic were used. About 1–2 mm explants were transplanted via trochar subcutaneously and allowed to grow for 1–2 months and then retransplanted into a different reporter transgenic. The growing tumors were observed in vivo with the OV100 small animal imaging system (Olympus Corp., Tokyo, Japan). Extirpated tumors were fixed in 4% paraformaldehyde and infiltrated with a 15–30% sucrose gradient, embedded in OCT, and sectioned on a cryostat. Select sections were counterstained with and without DAPI and subjected to dual fluorescence studies using rabbit anti-LYVE1 followed by Alexa Fluor 594-conjugated goat anti-rabbit secondary antibodies, respectively (Invitrogen, Waltham, MA, USA). As before, the sections were viewed with an Olympus Fluoview-1000 confocal scanning system (Olympus Corp., Tokyo, Japan).

### 4.11. Statistical Analysis

All the individual in vitro experiments were replicated 5 times. Within each experiment, 5 technical replicates were also conducted. The representative results depicted show either means or means ± standard deviations. All in vivo (murine) experiments were performed in groups of 10 mice. The illustrated photomicrographs depicting histopathology, fluorescence, and immunocytochemistry were representative of our typical results. All the stated or calculated differences imply differences in statistical significance, assessed by the two-tailed Student’s *t*-test and ANOVA.

## Figures and Tables

**Table 1.** Differential VEGF and endothelial haptotaxis.

Cell Line	VEGF	Vascular Haptotaxis	Lymphatic Haptotaxis

**Mary-X**	+++++ VEGF-C ++++ VEGF-D	+++++	+++++
**231**	+++ VEGF-C ++ VEGF-D	++	++
**MCF-7**	++ VEGF-C + VEGF-D	+	+
**HMS-1** *	− VEGF-C − VEGF-D	−−	−−

* HMS-1, derived from HMS-X, a human myoepithelial xenograft [13,14], devoid of blood vessels and lymphatics and expressing predictably low VEGF mRNA and endothelial haptotaxis activities.

## Data Availability

Both Mary-X and the other cell lines used in this study are available to any investigator upon request. All the data sets generated and used in the study are also available upon request.
